# Distributional Impacts of Tobacco Excise Taxes in Serbia

**DOI:** 10.1093/ntr/ntae175

**Published:** 2024-07-17

**Authors:** Jovan Zubović, Olivera Jovanović, Boban Nedeljković

**Affiliations:** Institute of Economic Sciences, Welfare Economics Department, Belgrade, Serbia; Institute of Economic Sciences, Welfare Economics Department, Belgrade, Serbia; Institute of Economic Sciences, Welfare Economics Department, Belgrade, Serbia

## Abstract

**Introduction:**

Numerous studies worldwide have investigated the impact of tobacco tax increase on household welfare, focusing on concerns regarding potential tobacco taxation regressivity and its effects on the poorest, whereas their scope was limited to the working population.

**Aims and Methods:**

To explore the effects of tax changes on household budgets, accounting for the retired population as well, we employed the modified Extended Cost–Benefit Analysis framework, assuming a 43.6% specific tax increase that fits the European Union (EU) Tobacco Tax Directive recommendation of minimum EUR 90 excise taxes per 1000 cigarettes. Our analysis encompassed changes in (1) tobacco expenditure (accounting for price elasticities by income groups: low-, middle-, and high-income), (2) medical costs linked to smoking-related diseases (utilizing relative risk of morbidity or mortality and smoking-attributable fractions), (3) years of working life (considering the years of working life lost among the working population), and (4) years of pension receipt (accounting for the years of retirement life lost due the premature death).

**Results:**

Under an assumed specific excise increase leading to a 22.4% retail tobacco price rise, the net gains in disposable household budgets would be 0.01% for high-income, 1.3% for middle-income, and 2.9% for low-income households.

**Conclusions:**

A tax increase would yield a progressive effect on income distribution, benefiting the most economically disadvantaged population thereby contributing to a more equitable income distribution.

**Implications:**

To effectively reduce tobacco consumption, subsequent smoking-related medical costs, and associated productivity and pension losses, it is recommended that Serbia implement a minimum 43.6% increase in the specific tobacco excise tax.

## Introduction

Tobacco use remains a major global health concern, with the World Health Organization (WHO) estimating that over eight million people die annually due to tobacco-related causes.^1^ Projections indicate that tobacco-related mortality and disability are expected to become the leading causes of premature death and disability worldwide by 2030. The Centers for Disease Control and Prevention (CDC) in the United States also highlights that 20%–40% of premature deaths could be prevented through lifestyle modifications such as smoking cessation and healthier dietary choices.^2^

Cigarette smoke contains over 7000 chemical compounds, with more than 250 posing health risks. Nearly 70 of those are known to cause cancer.^3^ Smoking is associated with various chronic noncommunicable diseases, and the risk escalates with prolonged smoking and increased cigarette consumption. Reducing smoking prevalence could significantly decrease global lung cancer incidence.^4^ Quitting smoking has several benefits for any smoker, regardless of individual smoking habits and history and corresponding individual-level risks; some of them are immediate, while others take years to manifest, according to the Centers for Disease Control and Prevention.^5^ The CDC confirms that nicotine level drops to zero after 24 h from quitting, the risk of heart attack drops sharply after 1 year, the risk of lung cancer is halved after 10 years, and most risks are fully eliminated after 20 years.

Most tobacco users worldwide live in low- and middle-income countries, where high smoking prevalence contributes to many premature deaths and tobacco-related diseases. Additionally, individuals with lower personal or family income are more susceptible to tobacco-related illnesses than their higher-income counterparts. As a result, tobacco use diverts spending from essential needs like food, clothing, education, and healthcare for lower-income households.^6^

Located in Southeastern Europe, Serbia is an upper-middle-income country grappling with a significant issue of tobacco consumption. The population of Serbia surpasses 6.7 million individuals, residing in around 2.6 million households as of 2022.^7^ On average, each household in Serbia consists of 2.55 people, while the average monthly income is approximately EUR 670 (annually EUR 8040).^7^ Although smoking prevalence has declined somewhat over time, it remains high at nearly 38% among adults, with men (approx. 1.13 million) just slightly outnumbering women (approx. 1.11 million) in smokers, according to the 2019 data.^8^ Given the substantial number of smokers, the Serbian population is vulnerable to developing smoking-related diseases, leading to increased medical expenditures and productivity losses due to illness and premature death. WHO estimates that over 1.2 million current smokers in Serbia could die prematurely if stronger tobacco control measures are not implemented.^9^ In 2016, more than 15 000 premature deaths were associated in Serbia due to smoking, with lung cancer and malignant diseases of the trachea being a prominent cause.^10^

Despite the adverse health and economic consequences of tobacco consumption, Serbia’s tobacco taxes are currently below the levels specified under the Association Agreement and the European Union (EU) Directive 2011/64/EU. This agreement commits Serbia to implementing excise duties on cigarettes at 60% of the average weighted retail price and not less than EUR 90 per 1000 cigarettes. Increasing tobacco taxes is aligned with the WHO Framework Convention on Tobacco Control (FCTC), which Serbia ratified in 2006. Both specific and ad valorem taxes are continuously applied to tobacco products in Serbia. The so-called “excise calendar” sets the specific excise semi-annual growth, followed by a price increase of RSD 10 (EUR 0.08). However, this growth is insufficient to effectively reduce tobacco consumption, as it does not keep pace with income growth, resulting in increased affordability of tobacco products.

This research focuses on assessing the potential impact of higher tobacco taxes in reducing the social and health costs associated with tobacco consumption. To achieve this, we utilize the partially modified extended cost–benefit analysis (ECBA) methodology,^11^ which considers the effects of decreased tobacco consumption due to increased prices. The analysis typically involves three main components: changes in the disposable household budget, reductions in tobacco-related medical expenses, and extension of working life years. In this study, we augment the ECBA by incorporating the impact of increased pension revenues resulting from extended years of life as the fourth component.

Various studies worldwide have examined the net impact of tobacco tax increases on household welfare, addressing concerns about potential tobacco tax regressivity and its impact on people experiencing poverty. The ECBA framework, often used in such analyses, aggregates costs and benefits to assess the medium- and long-term effects of projected tobacco tax increases. Tobacco tax increases, while inducing negative household budget shocks for smokers who maintain their consumption, can lead to reduced tobacco expenditures if smokers adjust their behavior in response to the price increase. As presented in the following paragraphs, research showed that at the aggregate level, the net effects of tax increases are positive across countries meaning a reduction in expenditures on tobacco and resulting tobacco-related health costs and loss in working life years. However, at the individual household level, the effects remain negative for smoking households, as their price elasticity is typically below one in absolute terms. In simpler terms, the quantity of tobacco products demanded by smoking households is not highly influenced by price changes. In other words, a price change will lead to a relatively small change in the amount of tobacco products demanded. This implies that smokers are less responsive to price fluctuations than other consumers.

Assessing the distributional effects of tobacco tax increases in low- and middle-income countries, the authors^12^ found negative direct effects on household budgets, which were offset by long-term gains from reduced medical expenses and extended working life. Similarly, a Mexican study^13^ yielded overall positive effects for low-, middle-, and high-income households, assuming a 58% retail price increase, with the highest net gains (over 4%) for the low-income group. These studies emphasize the importance of price elasticity in determining the effects on welfare, with higher price responsiveness leading to greater benefits for specific subgroups.

Positive and progressive effects on all-income groups have been observed in different countries, including China,^14^ Ukraine,^15^ Moldova,^16^ Vietnam,^17^ Peru,^18^ Argentina,^19^ and Brazil.^20^ In the Western Balkan region, previous cost–benefit analyses for Bosnia and Herzegovina,^21^ and Montenegro^22^ have demonstrated progressive effects on income distribution, resulting in substantial welfare improvements for low-income groups. However, such research has been lacking in Serbia, and this study aims to address this gap and contribute to the body of research on tobacco tax increases in the Western Balkan region. Moreover, findings go beyond supporting the progressivity hypothesis and provide added value by extending the ECBA model to include the benefits of the extended period of pension revenues resulting from decreased smoking prevalence.

## Materials and Methods

We used an extended cost–benefit analysis (ECBA) model with the formula below to estimate the net effects of increased tobacco taxes.


$$\begin{array}{l} \begin{array}{lllll} Net\;income\;effects=\; & Change\;in\;tobacco\;expenditure\left(A\right)\; & +Change\;in\;medical\;expenses\left(B\right)\; & +Change\;in\;years\;of\;productive\;life\left(C\right)\; & +Change\;in\;years\;oflife\;in\;pension\left(C+\right)\; \end{array} \end{array}$$ (1)

where

(A) = increase in tobacco expenditures after the tax increase at the household level,

(B) = decrease of direct medical expenses needed for tobacco-related medical treatments,

(C) = additional income households can earn by increasing their productive life years, and

(C+) = additional pensions households can receive by increasing years of life.

The net effects were calculated at the household level. The difference from the calculation at the aggregate national level was in medical expenses (B), which include both the direct governmental health costs and the out-of-pocket costs used in the household calculations. The estimation of the effects was calculated using a scenario of a 43.6% increase in the specific excise tax, which complies with the EU Tobacco Tax Directive recommendation of 90 EUR in excise taxes per 1000 cigarettes.

### Changes in Tobacco Expenditures

Data used for the estimation of changes in tobacco expenditures are price elasticities of quantity demanded by income group (tercile), quantity, and spending on cigarettes by household and individual and total income (spending), and tobacco tax structure (decomposed retail price).

Changes in tobacco expenditures are calculated using the formula^23^:


$$\begin{array}{l} \frac{E_{C_{0}}}{E_{T_{0}}}\left(\left(1+\;\%\;\Delta p\right)\left(1+\varepsilon_{p}\;\%\;\Delta p\right)-1\right) \end{array}$$ (2)

where

$E_{C_{0}}$ = spending on cigarettes (tobacco),

$E_{T_{0}}$ = total income,

Δp = change in price, and

$\varepsilon_{p}$ = tobacco price elasticity.

To estimate the change in the retail price of cigarettes resulting from a 43.6% change in specific excise tax, we used the formula:


$$\begin{array}{l} p_{cig}=p_{not}+\tau_{esp}+\;\;\;p_{cig}\times \tau_{eav}+p_{cig}\times \tau_{vat} \end{array}$$ (3)

where

*p*_cig_ = price of cigarettes,

*p*_not_ = price net of tax,

*t*_esp_ = specific excise,

*t*_eav_ = ad valorem excise, and

*t*_vat_ = value-added tax.

Hence, the new retail price is:


$$\begin{array}{l} p_{cig}^{*}=\frac{t}{1-\tau_{eav}-\tau_{vat}}\times \tau_{esp\;\;\;}+\;\;\;\;\;p_{cig} \end{array}$$ (4)

where

*t* = total tax.

For this study, we assumed full pass-through from tax increase to price increase—that is, the entire increase in tobacco tax will be transmitted to an increase in prices ($p_{not}$ remains constant). The current value-added tax (VAT) rate in Serbia is 20%. The ad valorem rate is 33%, and the average specific tax in 2019 was RSD 70.75 per pack. There is no tiered tax structure. The initial estimation was made using the weighted average price (WARP), published once a year by the Tobacco Administration Office, which was RSD 274.24 in 2019. The increase of 43.6% in the specific tax would lead to a 22.35% increase in retail price (see Table 1 for details on cigarette price structure changes).

**Table 1.** Change in the Structure of Cigarette Price in Serbia with 43.6% Increase of Specific Excise Tax (2019)

	*p*_cig_	*p*_not_	*t*_esp_	*t*_eav_	*t*_vat_	Tax share	Excise share
Initial	274.24	67.28	70.75	90.50	45.71	75.47%	58.80%
43.6% increase	335.53	67.28	101.60	110.72	55.92	79.95%	63.28%
Δ	22.35%		43.6%				

*Source: Tobacco administration office in Serbia, authors’ calculations*.

*p*_cig_ = price of cigarettes; *p*_not_ = price net of tax; *t*_esp_ = specific excise; *t*_eav_ = ad valorem excise; *t*_vat_ = value-added tax.

To estimate the average change in tobacco expenditure by household, price elasticities by income group were applied to two different price changes. Using prevalence rates by income groups (see Table 2) from the Household Budget Survey (HBS), we calculated the total number of smokers by income group. According to a 2019 report,^24^ price elasticities for tobacco products in Serbia differ among income groups. Income groups were constructed based on total household expenditures (a proxy for income) per capita. Given the relatively small sample size, three income groups were created using split by terciles: low-income, middle-income, and high-income. After dividing the sample into three income groups, prevalence elasticity was estimated using a logit model and conditional demand (intensity) elasticity using the Deaton model (see Supplementary file, Formula S1). The price elasticities are displayed in Table 2.

**Table 2.** Smoking Prevalence Rates and Price Elasticities by Income Groups

	Smoking prevalence rates^a^			Price elasticity^b^		
	Hand-rolled cigarettes	Manufactured cigarettes	Total	Lower^*^ bound	Middle^*^ bound	Upper^*^ bound
Low income	8.2%	36.0%	41.0%	–0.934	–1.076	–1.218
Middle income	5.8%	32.6%	36.9%	–0.496	–0.631	–0.766
High income	5.1%	30.4%	34.9%	–0.179	–0.220	–0.261
Total	6.4%	33.1%	37.7%			

*Sources* ^a^: study^6^; ^b^ paper^8^.

^*^The values of the lower, middle, and upper bounds are derived using the data (Table 8.3) from the study.^24^ As the calculation included the correction for the associated variance, the middle bound represents the most likely value, while the lower and upper bound represent the lowest and the highest possible value, respectively.

Of note, the estimated elasticities pertain to spending only on manufactured cigarettes, whose share in the market is 83.1%.^8^ Given that a very high share of manufactured cigarettes (97.6%) is sold on the licit market,^25^ their consumption is a good proxy for estimating the impact of changes in the specific excise tax rate on total spending on tobacco products.

### Change in Tobacco-Related Direct Medical Expenses

The effect of reducing medical expenses after the tax-induced price increase in cigarettes yields positive income gains for all-income groups, as the price shock encourages a reduction in smoking and, hence, a drop in tobacco-related medical expenses. Households can then benefit from higher disposable income, as they are no longer burdened by those medical bills.

Following the assumption of a 43.6% specific excise increase, we estimated the change in tobacco-related direct medical expenses in two stages for all-tobacco-attributable diseases (based on information on relative risk from the US Department of Health and Human Services), by gender, age group, and type of illness.

The first stage was a calculation of the smoking-attributable fraction (SAF) of medical expenses. The data required included:

List of smoking-related ICD codes (Supplementary File, Table A1).

Public medical expenditure for the treatment of smoking-related diseases from the Republic Fund of Health Insurance by age, gender, and ICD code in Serbia in 2019 (Supplementary File, Table A2).

Estimated out-of-pocket medical expenditures for the treatment of smoking-related diseases (Supplementary File, Table A9), and

Relative risk (RR) of mortality or morbidity by ICD code from smoking and smoking prevalence to calculate smoking-attributable fraction (SAF).

Data were applied for each age, gender group, and disease type, both for current smokers and former smokers:


$$\begin{array}{l} SAF_{ag}=\frac{Pe_{ca}*\left(RR_{c}-1\right)+\;\;\;Pe_{fa}\times \left(RR_{f}-1\right)}{Pe_{ca}*\left(RR_{c}-1\right)+\;\;\;Pe_{fa}\times \left(RR_{f}-1\right)+1}\times 100\;\%\; \end{array}$$ (5)

where

*SAF*_ag_ = smokg-attributable fraction by age and gender,

$Pe_{ca}$ = prevalence of current smokers in age group *a*,

$Pe_{fa}$ = prevalence of former smokers in age group *a*,

$RR_{c}$ = relative risk mortality or morbidity by disease for current smokers, and

$RR_{f}$ = relative risk mortality or morbidity by disease for former smokers.

Calculation of the change in tobacco-related medical expenses follows:

(1) Define the list of smoking-related diseases by ICD-10 codes (details in Supplementary File, Table A1).
(2) Obtain data on medical expenses and calculate SAF and smoking-attributable spending for each income group.
(3) Calculate the change in medical expenditures by income group by:

- Using mortality RRs as the proxy for morbidity RR^4^ (for details, see Supplementary File, Table A2);
- Applying the formula for calculation of SAF^26^ on the smoking prevalence rates for current and former smokers (SAF is the same for current and former smokers) and RRs for each smoking-attributable disease and by gender and age group (Supplementary File, Table A4); and
- Applying SAF on data from Table 2.

The effect on income from reducing medical expenditures was calculated by:


$$\begin{array}{l} \Delta SAHE_{i}=\left(\varepsilon_{p}*\;\%\;p\right)\times \frac{E_{MC_{0}}}{E_{T_{0}}} \end{array}$$ (6)

where $E_{MC_{0}}\;$ represents medical spending on treatment of tobacco-related diseases, *i* stands for income group, and the other values in the equation are the same as in Equation (2).

Total healthcare expenditures in Serbia were estimated at USD 641 per capita in 2019, according to the World Bank (2022)^27^, resulting in a total of USD 4.45 billion, which accounted for 8.6% of the country’s GDP in 2019. Out-of-pocket payments constituted a significant portion of these expenditures, representing 40% of the health expenditures. To estimate smoking-related costs, we relied on the Republic Fund for Social Protection (RFSP) data on public expenditure (Supplementary File, Table A2), accounting for 60% of the total spending. We rescaled this amount to 40% and then allocated these costs to three income groups, following the distribution of out-of-pocket medical expenditures among the groups obtained from the HBS (Supplementary File, Table A8). Even though the HBS includes data on medical expenditure, we did not utilize them due to a well-known tendency of underreporting in such surveys. Instead, we used the official RFSP data for more accurate estimates (Supplementary File, Table A9).

### Additional Income Earned From Increased Years of Productive Life

In this section, we estimated the value of additional income that could be earned by all-household members resulting from an increase in the specific excise tax by 43.6%. Changes in the years of working life lost (YWLL) were estimated by income groups and five-year age cohorts (Formula (7)). Data required for calculation include:

Smoking-attributable death events (SAF × total number of deaths from smoking-related diseases) and years of life lost among the working population.

Data on the number of deaths were extracted from the Institute for Public Health database (Republic Fund of Health Insurance, 2022). SAF is calculated using RR estimates, as explained above. Additionally, RR rates from WHO databases^26^ and available estimates for Eastern European countries were used for robustness checks.^28^


$$\begin{array}{lll} & Ef\;fect\;on\;income\;from\;reducing\;YWLL\;\; & \;=\;((\varepsilon_{p}\times \;\%\;p\;\;\;\times \;YWLL_{i})\times \frac{HIi}{E_{T_{0}}}) \end{array}$$ (7)

where,

$\varepsilon_{p}$ = price elasticity per income group,

%*p* = percentage change in price,

*YWLL*_*i*_ = number of years of working life lost per smokers’ household per income group, and

$\frac{HIi}{E_{T_{0}}}$ = share of the household income in the total household budget.

Of note, in this study, we used HBS data, where total expenditure is seen as a proxy for income, assuming that the ratio equals one. If detailed information on income and spending per household had been accessible, the ratio might have varied.

To estimate the increase in working years by income group, the total tobacco-attributed years of life lost were distributed across income groups proportionately to the number of households that consume tobacco per income group.

Additional income earned was estimated using the following steps:

Identifying the number of deaths among the working population by age group and ICD10 (Supplementary File, Table A5),

Estimate YWLL by age group using SAF (see Supplementary File, Tables A4 and A6).

Determining income by age cohorts using HBS data,

Calculating the effects on income using Equation (7).

### Additional Income Earned From Increased Years of Pension Receipt

Similar to the increase in income from increased years of productive life, we estimated the increase in pensions available due to the increase in years of life, considering the average life expectancy in Serbia of 75 years.^29^

The effect on income from decreasing years of pension life lost was calculated as:


$$\begin{array}{l} YPLL=((\varepsilon_{p}\times \;\%\;p\times YPLL_{i})\times \frac{HIi}{E_{T_{0}}})\;\;\; \end{array}$$ (8)

where

$\varepsilon_{p}$ = price elasticity per income group,

%*p* = percentage change in price,

*YPPL*_*i*_ = number of years of pension life lost per smokers’ household per income group, and

$\frac{HIi}{E_{T_{0}}}$ = share of the household income in the total household budget.

The effect on income from reducing YWLL was calculated using the following formula:


$$\begin{array}{l} (\varepsilon_{p}\times \;\%\;p\;\;\;\times \;(YRLL+YWLL_{i})\;\times \;\frac{HIi}{E_{T_{0}}} \end{array}$$ (9)

where

*YRLL* = years of retirement life lost.

## Results

Complying with the aim of the study, we first calculated the effects for the A, B, C and C+ parts of the model, and subsequently, the net gains accounting for all-parts. Thus, the results presented below follow the order of analyses.

### Part A

**Figure 1.**
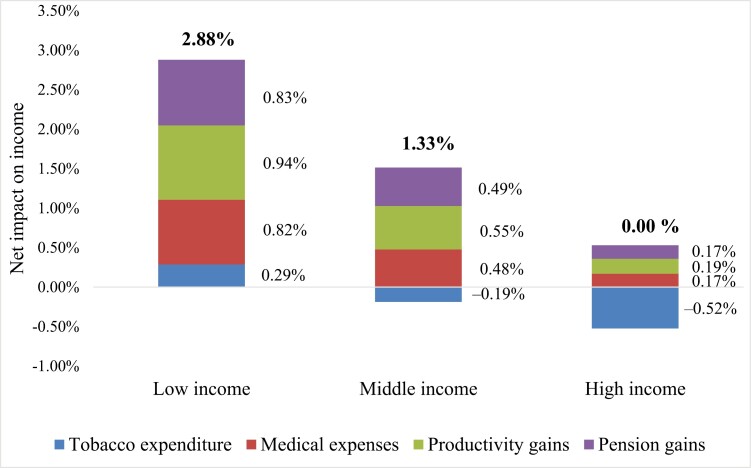
Impact of 43.6% specific tobacco excise increase on net income due to changes in tobacco expenditure, medical expenses, productivity gains, and pension gains (middle bound values). Source: Authors’ calculation.

As displayed in Figure 1, a 22.4% cigarette price increase would have a positive effect on the low-income group in terms of decreased tobacco expenditure. In contrast, the high-income group would experience a slight loss. Implementing this increase would have a progressive effect, meaning lower affordability, reduced consumption, and more resources available for other beneficial spending.

### Part B

Increasing tobacco taxes could further enhance the progressive effect on income through the resulting reduction of tobacco-related medical expenditures. The analysis (see also Figure 1) demonstrated positive effects on income in all-groups, with the greatest gain observed in the low-income group, further confirming the progressive effect of tax increases regardless of the elasticity and SAF assumption. The highest benefits in the poorest group are derived from higher responsiveness to price changes and a lower income base, similar to the changes in tobacco expenditures (Part A). The poorest population group would have more resources after the tax increase, as the reduced prevalence and quantity consumed would lower the incidence of smoking-related diseases and, subsequently, the spending to treat them.

### Part C

Apart from resulting in a decline in smoking prevalence and a reduction of expenses for treating smoking-related diseases, the increase in tobacco taxes would also decrease the number of smoking-attributable deaths. The positive effects result from the higher earnings associated with the lower number of years of working life lost (YWLL) or increased number of years at work. As displayed in Figure 1, there is a positive income gain for all-income groups. The results demonstrated that all-three income groups would gain additional income due to the lower number of YWLL, with the highest increase in the low-income group of nearly 1%.

### Part C+

As mentioned previously, in this study, we introduced the assumption that change in household income also occurs for extended periods of time, including pension income. The current average life expectancy in Serbia is 75 years (approximately ten years after the retirement age). In estimating the gains from years of retirement life lost (YRLL), we reduced their effect to 42.3%, accounting for the ratio between average pension and average wage.

Figure 1 shows the change in disposable household income resulting from extended periods of pension receipt, which derives from reduced smoking prevalence. Similar to the former parts of the model, the observed effects were positive, pointing to an overall positive gain for all-income groups, with the highest gains for the low-income group.

### Net Gains

**Figure 2.**
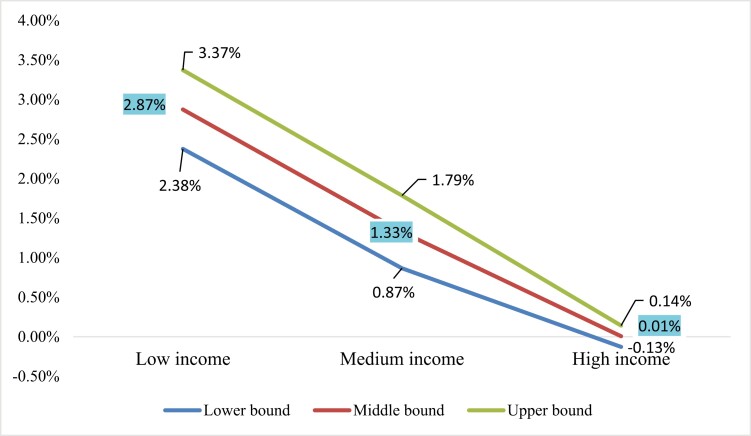
Net gains resulting from A, B, C, and C+ components. *Source*: authors’ calculation.

Finally, to estimate the net gains, we summed up the changes in consumption, medical costs, productivity, and pensions. The middle-bound values are displayed in Figure 2 (for lower-bound and upper-bound, see Supplementary File, Table A12). The highest gains in disposable income were projected for the low-income group, thus confirming the progressivity of tobacco tax increases. The simulated net income gain magnitude ranged from 2.38% to 3.37% in this group. It was shown to be lower but also positive for the middle-income group, ranging between 0.87% and 1.79%, while varying between negative (–0.13%) and positive (0.14%) for the high-income group. For comparison, we employed the same analysis by excluding the pensions from the model, which indicated that the net gains for low-income (ranging between 1.65% and 2.43%) and middle-income groups (ranging between 0.48% and 1.19%) would be comparably lower, while the net gain for a high-income group would be negative, falling in the range from –0.27% to –0.06% (see Supplementary File, Table A11).

## Discussion

This study estimated the impacts of tobacco taxes in Serbia using an extended cost-benefit analysis. The main goal of this research was to determine the gains at the household level that could be obtained from the reduction in tobacco consumption resulting from the 43.6% specific excise tax increase. To estimate the excise, increase net effects on the income, we accounted for the changes in (A) tobacco expenditure, medical expenses related to smoking-attributable diseases (B), income resulting from extension in years of productive life (C), and additionally, income generated through an extended period of pension revenues as a result of reduced smoking prevalence (C+). The simulated net effect for summing up the components A, B, and C was shown to range in magnitude from 1.65% to 2.43% (EUR 94–139 annually) in the low-income group while ending up with a loss between –0.06% and –0.27% for the high-income group. The inclusion of income change resulting from pensions (C+ component) yielded even more positive effects, indicating a net income gain of 2.8% (EUR 164 annually based on 2019 data) for the low-income group and 0.01% for the high-income group. If we apply the net gain of 2.8% to more recent data from 2022 (HBS), in which income has increased by 17.2%, the nominal annual net gain would reach EUR 192. In an alternative scenario in which the specific excise is increased by 100% instead of 43.6%, resulting in a price increase of 51.3%, the increase in disposable income would be 7.2% for the low-income group, 3.4% for the middle-income group, and 0.1% for the high-income group (see Supplementary Table A13).

Looking back at the particular components comprising the model, we could notice the differences in their effects across low-, middle-, and high-income groups. According to our scenario, low-income households will likely have disposable budget gain from reduced tobacco expenditure itself, which will be further increased by the cumulative effects of the other three components. For middle-income households, a slight loss resulting from higher tobacco expenditure is likely to be outweighed by gains in reduced medical expenses and an increase in years of productive life and pension receipt, whereby each of these three components itself surpasses the loss resulting from the tobacco price increase. This effect is somewhat different in the high-income households. Neither of the three components outweighs the household budget loss from increased tobacco expenditure in this group due to their inelasticity. However, these three components cumulatively foreclose the negative effect, eventually resulting in the disposable budget in high-income households remaining unaffected.

The results of our study indicated that increasing tobacco taxes can contribute to a more equitable distribution of income, with the highest benefits observed in the most economically vulnerable population segment. These results are fairly aligned with findings from other countries, including Bosnia and Herzegovina and Montenegro, located in the Western Balkan region,^21,22^ and provide additional evidence of progressive effects in tobacco taxation. One of the advantages of the study is that it originates from Serbia and thus expands the knowledge on tobacco taxation effects in low- and middle-income countries. It is possible to directly compare the results with other studies that employed the traditional ECBA framework. For example, the study from Mexico,^13^ assuming a retail price increase of 58% to reach 75% tax coverage, indicated a net gain of 4% in the household budget for low-income households. On the other hand, the study from Montenegro^22^ that used a comparable 50% tax increase showed that the net gain for low-income households falls between 1.6% and 1.8%, which is quite similar to our findings based on the three traditional components. However, the inclusion of pension revenues significantly increased the net gain in our analysis, and hence, we believe that the modified model we used provides a more accurate estimation, while other studies that relied on the traditional model might have underestimated the net effect.

In this study, we utilized data from various comparable sources, disaggregated by income level, gender, and type of disease, to ensure the most accurate estimations. Nevertheless, we must acknowledge certain limitations. All-estimations were made solely for manufactured cigarettes; however, their share in the Serbian tobacco market is 83.1%, which provides a substantial basis for drawing general conclusions. It is important to note that when cigarette prices increase, there may be some substitution from manufactured to hand-rolled cigarettes, which we were unable to account for due to the lack of data on cross-price elasticity between manufactured cigarettes and hand-rolled cigarettes, as well as between different market price segments of manufactured cigarettes.

In conclusion, our findings go beyond supporting the progressivity hypothesis and provide added value by extending the ECBA model. To the best of our knowledge, this was the first study that accounted for the income generated through an extended period of pension revenues resulting from decreased smoking prevalence. Supplementing the traditional ECBA model underscored the essential role of pension revenues in the household budget and, consequently, in the income changes derived from tobacco price and consumption changes. Given this, future studies should employ this approach to provide a more comprehensive and accurate estimation of the total net effects on household income, including the impact of change in presenteeism and absenteeism resulting from a change in smoking behavior. Lastly, considering the results, we need to make a policy recommendation: Serbia should implement a minimum of 43.6% increase in specific tax to reduce tobacco consumption effectively, subsequent smoking-related medical costs, and associated productivity and pension losses. Additional research is recommended to extend the scope of the study so as to include hand-rolled cigarettes and the segmentation of the cigarette market to at least three price categories.

## Supplementary material

Supplementary material is available at *Nicotine and Tobacco Research* online.

ntae175_suppl_Supplementary_File

## Data Availability

Data are available upon reasonable request.
