# Supplementary material for: Distributional Impacts of Tobacco Excise Taxes in Serbia
Source: Nicotine Tob Res. 2024 Jul 17;27(1):106–13. doi: 10.1093/ntr/ntae175 (PMC11663806; doi:10.1093/ntr/ntae175)
Supplement: ntae175_suppl_Supplementary_File [file ntae175_suppl_supplementary_file.docx]

**Supplementary file**

**Formula S1.** Total elasticity (Vladisavljevic & Zubovic, 2019)

𝜉𝑝_ig_ = 𝜉𝑝_ig_1 + (1 + 𝜉𝑝_ig_1) ∗ 𝜉𝑝_ig_2

where:

𝜉𝑝_ig_1 = prevalence elasticity;

𝜉𝑝_ig_2 = conditional demand (intensity) elasticity;

𝜉𝑝_ig_ = total elasticity, if all the elasticities are expressed as percentages; and

ig = income group.

**Table A1.** **Causes of smoking-related death according to International Classification of Diseases (ICD-10)**

| **Malignant Neoplasms** |  |
| --- | --- |
| Upper aerodigestive tract | C00-C14, C15, C32 |
| Stomach | C16 |
| Pancreas | C25 |
| Trachea, bronchus | C33–C34 |
| Cervix uteri | C53 |
| Kidney, other urinary | C64–C65 |
| Urinary bladder | C67 |
| Acute myeloid leukemia | C92 |
| **Cardiovascular Diseases** |  |
| Other heart disease, all ages | I00–I09, I26–I51 |
| Ischemic heart disease | I20–I25 |
| Cerebrovascular diseases | I60–I69 |
| Arterial disease | I70, I71, I72-I78 |
| **Respiratory Diseases** |  |
| Other respiratory diseases | J10–J18 |
| Chronic obstructive pulmonary disease | J40-J42, J43, J44 |

Source: WHO (2011)

**Table A2.** **Public medical expenditure for treatment of smoking-related diseases in Serbia, 2019 (RSD)**

| **Gender** | **ICD-10 Code** | **Number of interventions*** | **0-35** | **36-65** | **65+** | **Total** |
| --- | --- | --- | --- | --- | --- | --- |
| F | C00-C14 | 1,451 | 1,750,764 | 38,581,378 | 25,148,780 | 65,480,922 |
| M | C00-C14 | 2,953 | 4,108,928 | 97,661,362 | 74,614,872 | 176,385,162 |
| F | C15 | 255 | 6,186 | 8,962,080 | 8,897,599 | 17,865,865 |
| M | C15 | 755 | 763,338 | 44,864,758 | 36,361,368 | 81,989,465 |
| F | C25 | 1,318 | 1,380,339 | 61,493,143 | 91,628,937 | 154,502,420 |
| M | C25 | 1,847 | 1,480,776 | 88,938,850 | 108,877,824 | 199,297,449 |
| F | C32 | 800 | 13,717 | 20,175,951 | 17,357,128 | 37,546,796 |
| M | C32 | 3,124 | 59,681 | 130,752,651 | 120,960,322 | 251,772,653 |
| F | C33-C34 | 7,230 | 4,129,165 | 464,662,880 | 457,170,232 | 925,962,277 |
| M | C33-C34 | 12,591 | 5,493,210 | 668,368,634 | 806,206,380 | 1,480,068,224 |
| F | C53 | 7,251 | 15,781,789 | 218,431,052 | 67,852,729 | 302,065,570 |
| M | C53 | 13 | 499 | 296,433 | 14,425 | 311,357 |
| F | C64-C65 | 1,570 | 1,981,168 | 31,253,242 | 31,594,077 | 64,828,487 |
| M | C64-C65 | 2,482 | 8,703,843 | 59,592,223 | 57,052,852 | 125,348,918 |
| F | C67 | 3,688 | 431,802 | 56,375,030 | 90,392,395 | 147,199,226 |
| M | C67 | 6,914 | 568,332 | 149,649,777 | 269,642,353 | 419,860,462 |
| F | I20-I25 | 48,970 | 6,652,761 | 676,820,343 | 1,189,822,324 | 1,873,295,429 |
| M | I20-I25 | 72,496 | 26,368,505 | 2,254,413,747 | 2,167,630,056 | 4,448,412,307 |
| F | I00-I09; I26-I28; I29-I51 | 90,190 | 108,782,105 | 681,227,977 | 1,527,873,983 | 2,317,884,064 |
| M | I00-I09; I26-I28; I29-I51 | 93,853 | 204,058,107 | 1,297,717,242 | 1,847,840,322 | 3,349,615,671 |
| F | I60-I69 | 35,105 | 38,513,008 | 782,103,051 | 1,024,193,608 | 1,844,809,667 |
| M | I60-I69 | 37,555 | 53,366,377 | 709,937,426 | 928,379,304 | 1,691,683,106 |
| F | I70 | 5,221 | 419,766 | 48,131,396 | 91,509,610 | 140,060,772 |
| M | I70 | 8,627 | 900,548 | 114,873,365 | 168,627,732 | 284,401,645 |
| F | I71 | 1,649 | 1,152,232 | 54,211,750 | 133,626,818 | 188,990,799 |
| M | I71 | 5,426 | 16,752,557 | 306,899,412 | 418,640,488 | 742,292,457 |
| F | I72-I78 | 5,660 | 2,780,623 | 33,599,807 | 60,263,141 | 96,643,571 |
| M | I72-I78 | 4,875 | 8,982,455 | 82,958,710 | 124,495,203 | 216,436,367 |
| F | J10-J11; J12-J18 | 118,953 | 256,785,191 | 2,261,217,971 | 4,415,345,350 | 6,933,348,512 |
| M | J10-J11; J12-J18 | 135,860 | 386,489,151 | 3,821,963,927 | 4,891,525,291 | 9,099,978,369 |
| F | J40-J42; J43 | 6,284 | 2,857,039 | 10,913,366 | 10,538,317 | 24,308,722 |
| M | J40-J42; J43 | 6,355 | 5,385,299 | 21,422,125 | 18,897,081 | 45,704,505 |
| F | J44 | 18,404 | 2,099,507 | 123,909,246 | 168,155,661 | 294,164,413 |
| M | J44 | 23,608 | 4,932,305 | 131,294,149 | 251,639,210 | 387,865,664 |
|  |  | **773,333** | **1,173,931,074** | **15,553,674,453** | **21,702,775,767** | **38,430,381,294** |

Source: Republic Fund for Social Protection of Serbia (2022)

*Note: The number of interventions includes the number of health activities (or interventions) related to the appropriate ICD–10 Codes.

**Table A3.** **Risk rates by ICD-10 codes**

| Disease category (ICD–10 code) | Men | | Women | |
| --- | --- | --- | --- | --- |
|  | Current smoker | Former smoker | Current smoker | Former smoker |
| Malignant neoplasms |  |  |  |  |
| Lip, oral cavity, pharynx (C00–C14) | 10.89 | 3.40 | 5.08 | 2.29 |
| Esophagus (C15) | 6.76 | 4.46 | 7.75 | 2.79 |
| Stomach (C16)* | 1.96 | 1.47 | 1.36 | 1.32 |
| Pancreas (C25) | 2.31 | 1.15 | 2.25 | 1.55 |
| Larynx (C32) | 14.60 | 6.34 | 13.02 | 5.16 |
| Trachea, lung, bronchus (C33–C34) | 23.26 | 8.70 | 12.69 | 4.53 |
| Cervix uteri (C53) | n/a | n/a | 1.59 | 1.14 |
| Kidney and renal pelvis (C64–C65) | 2.72 | 1.73 | 1.29 | 1.05 |
| Urinary bladder (C67) | 3.27 | 2.09 | 2.22 | 1.89 |
| Acute myeloid leukemia (C92.0)* | 1.86 | 1.33 | 1.13 | 1.38 |
| Cardiovascular diseases |  |  |  |  |
| Coronary heart disease (I20–I25) |  |  |  |  |
| Persons 35–64 years of age | 2.80 | 1.64 | 3.08 | 1.32 |
| Persons ≥65 years of age | 1.51 | 1.21 | 1.60 | 1.20 |
| Other heart disease (I00–I09, I26–I28, I29–I51) | 1.78 | 1.22 | 1.49 | 1.14 |
| Cerebrovascular disease (I60–I69) |  |  |  |  |
| Persons 35–64 years of age | 3.27 | 1.04 | 4.00 | 1.30 |
| Persons ≥65 years of age | 1.63 | 1.04 | 1.49 | 1.03 |
| Atherosclerosis (I70) | 2.44 | 1.33 | 1.83 | 1.00 |
| Aortic aneurysm (I71) | 6.21 | 3.07 | 7.07 | 2.07 |
| Other arterial disease (I72–I78) | 2.07 | 1.01 | 2.17 | 1.12 |
| Respiratory diseases |  |  |  |  |
| Influenza, pneumonia (J10–J11, J12–J18) | 1.75 | 1.36 | 2.17 | 1.10 |
| Bronchitis, emphysema (J40–J42, J43) | 17.10 | 15.64 | 12.04 | 11.77 |
| Chronic airways obstruction (J44) | 10.58 | 6.80 | 13.08 | 6.78 |
| * Data on medical expenses in Serbia not available | | | | |

Source: US Department of Health and Human Services (2014)

**Table A4.** **Smoking-attributable fraction in Serbiain 2019 , by ICD-10 code, smoking status and age**

|  |  | **Current smokers** | | | | | **Former smokers** | | | | |
| --- | --- | --- | --- | --- | --- | --- | --- | --- | --- | --- | --- |
| **Gender** | **ICD-10 code** | **35-44** | **45-54** | **55-64** | **65-74** | **75+** | **35-44** | **45-54** | **55-64** | **65-74** | **75+** |
| F | C00-C14 | 60.5% | 61.3% | 59.8% | 39.1% | 27.0% | 25.4% | 21.8% | 27.0% | 35.1% | 30.7% |
| M | C00-C14 | 81.2% | 77.2% | 76.5% | 67.0% | 60.2% | 39.5% | 55.2% | 65.6% | 71.6% | 62.6% |
| F | C15 | 71.4% | 72.0% | 70.8% | 51.1% | 37.7% | 35.6% | 31.2% | 37.7% | 46.8% | 41.9% |
| M | C15 | 73.6% | 68.6% | 67.8% | 56.7% | 49.4% | 29.6% | 44.3% | 55.2% | 61.9% | 52.0% |
| F | C25 | 32.7% | 33.4% | 32.1% | 17.0% | 10.5% | 9.7% | 8.1% | 10.5% | 14.7% | 12.3% |
| M | C25 | 35.5% | 30.2% | 29.4% | 20.6% | 16.2% | 7.7% | 13.6% | 19.6% | 24.4% | 17.6% |
| F | C32 | 82.0% | 82.4% | 81.5% | 65.6% | 52.4% | 50.2% | 45.3% | 52.4% | 61.6% | 56.8% |
| M | C32 | 86.1% | 82.9% | 82.4% | 74.5% | 68.5% | 48.4% | 63.9% | 73.3% | 78.4% | 70.7% |
| F | C33-C34 | 81.4% | 81.9% | 80.9% | 64.7% | 51.4% | 49.2% | 44.3% | 51.4% | 60.7% | 55.8% |
| M | C33-C34 | 90.9% | 88.7% | 88.3% | 82.5% | 77.9% | 60.2% | 74.1% | 81.6% | 85.4% | 79.6% |
| F | C53 | 17.8% | 18.2% | 17.3% | 8.3% | 5.0% | 4.6% | 3.8% | 5.0% | 7.1% | 5.9% |
| M | C53 | 0.0% | 0.0% | 0.0% | 0.0% | 0.0% | 0.0% | 0.0% | 0.0% | 0.0% | 0.0% |
| F | C64-C65 | 9.4% | 9.7% | 9.1% | 4.2% | 2.4% | 2.2% | 1.9% | 2.4% | 3.5% | 2.9% |
| M | C64-C65 | 44.2% | 38.3% | 37.5% | 27.2% | 21.8% | 10.7% | 18.5% | 26.0% | 31.7% | 23.5% |
| F | C67 | 33.9% | 34.6% | 33.3% | 17.7% | 11.1% | 10.2% | 8.5% | 11.1% | 15.3% | 12.9% |
| M | C67 | 51.4% | 45.4% | 44.5% | 33.3% | 27.1% | 13.8% | 23.3% | 31.9% | 38.3% | 29.2% |
| F | I20-I25 | 42.6% | 43.4% | 41.9% | 8.6% | 5.2% | 14.2% | 11.9% | 15.2% | 7.4% | 6.1% |
| M | I20-I25 | 44.8% | 39.0% | 38.1% | 10.0% | 7.6% | 11.0% | 18.9% | 26.5% | 12.1% | 8.4% |
| F | I00-I09; I26-I28; I29-I51 | 15.3% | 15.7% | 14.9% | 7.0% | 4.2% | 3.8% | 3.2% | 4.2% | 6.0% | 4.9% |
| M | I00-I09; I26-I28; I29-I51 | 25.5% | 21.2% | 20.6% | 13.9% | 10.7% | 4.9% | 8.9% | 13.2% | 16.7% | 11.7% |
| F | I60-I69 | 51.3% | 52.1% | 50.6% | 6.7% | 4.0% | 19.0% | 16.1% | 20.3% | 5.7% | 4.7% |
| M | I60-I69 | 48.1% | 42.1% | 41.2% | 11.0% | 8.4% | 12.3% | 20.9% | 29.1% | 13.3% | 9.2% |
| F | I70 | 22.1% | 22.7% | 21.6% | 10.6% | 6.4% | 5.9% | 4.9% | 6.4% | 9.1% | 7.6% |
| M | I70 | 38.5% | 32.9% | 32.2% | 22.8% | 18.0% | 8.7% | 15.2% | 21.7% | 26.8% | 19.6% |
| F | I71 | 68.6% | 69.3% | 67.9% | 47.8% | 34.6% | 32.7% | 28.5% | 34.6% | 43.5% | 38.7% |
| M | I71 | 70.4% | 65.1% | 64.3% | 52.8% | 45.5% | 26.5% | 40.5% | 51.3% | 58.2% | 48.0% |
| F | I72-I78 | 29.1% | 29.7% | 28.5% | 14.7% | 9.0% | 8.3% | 6.9% | 9.0% | 12.6% | 10.6% |
| M | I72-I78 | 30.2% | 25.4% | 24.7% | 17.0% | 13.2% | 6.2% | 11.0% | 16.1% | 20.2% | 14.4% |
| F | J10-J11; J12-J18 | 29.1% | 29.7% | 28.5% | 14.7% | 9.0% | 8.3% | 6.9% | 9.0% | 12.6% | 10.6% |
| M | J10-J11; J12-J18 | 25.8% | 21.4% | 20.8% | 14.1% | 10.9% | 5.0% | 9.0% | 13.3% | 16.9% | 11.9% |
| F | J40-J42; J43 | 83.2% | 83.6% | 82.7% | 67.5% | 54.5% | 52.3% | 47.4% | 54.5% | 63.6% | 58.8% |
| M | J40-J42; J43 | 89.4% | 86.9% | 86.4% | 79.9% | 74.7% | 56.0% | 70.7% | 78.9% | 83.1% | 76.6% |
| F | J44 | 82.6% | 83.1% | 82.2% | 66.6% | 53.5% | 51.3% | 46.4% | 53.5% | 62.6% | 57.8% |
| M | J44 | 82.2% | 78.4% | 77.8% | 68.6% | 61.9% | 41.2% | 57.0% | 67.2% | 73.1% | 64.3% |

**Table A5. Number of deaths in Serbia in 2019, by ICD-10 code and age group**

| **Gender** | **ICD-10 code** | **0-9** | **10-19** | **20-29** | **30-34** | **35-44** | **45-54** | **55-64** | **65-74** | **75+** |
| --- | --- | --- | --- | --- | --- | --- | --- | --- | --- | --- |
| F | C00-C14 |  |  |  |  | 8 | 9 | 12 | 38 | 54 |
| M | C00-C14 |  |  |  |  | 11 | 43 | 133 | 123 | 73 |
| F | C15 |  |  |  |  | 1 | 2 | 8 | 14 | 17 |
| M | C15 |  |  | 1 |  | 3 | 18 | 51 | 55 | 40 |
| F | C25 |  |  | 2 |  | 4 | 26 | 103 | 207 | 224 |
| M | C25 |  |  |  |  | 11 | 41 | 157 | 257 | 174 |
| F | C32 |  |  |  |  |  | 1 | 5 | 10 | 11 |
| M | C32 |  |  |  | 1 |  | 18 | 94 | 141 | 87 |
| F | C33-C34 |  |  |  | 1 | 24 | 124 | 484 | 638 | 348 |
| M | C33-C34 |  |  |  | 5 | 27 | 220 | 1,076 | 1,620 | 682 |
| F | C53 |  |  | 8 | 4 | 26 | 76 | 129 | 106 | 108 |
| M | C53 |  |  |  |  |  |  |  |  |  |
| F | C64-C65 |  |  | 1 |  | 3 | 12 | 30 | 45 | 38 |
| M | C64-C65 |  |  |  | 1 | 8 | 19 | 70 | 93 | 96 |
| F | C67 |  |  |  |  | 2 | 10 | 22 | 70 | 93 |
| M | C67 |  |  |  |  | 1 | 17 | 97 | 192 | 272 |
| F | I20-I25 |  | 1 |  | 1 | 11 | 72 | 240 | 772 | 3,150 |
| M | I20-I25 |  |  | 2 | 9 | 80 | 284 | 860 | 1,425 | 2,321 |
| F | I00-I09; I26-I28; I29-I51 | 2 | 7 | 9 | 3 | 38 | 136 | 531 | 1,728 | 10,331 |
| M | I00-I09; I26-I28; I29-I51 | 2 | 10 | 16 | 24 | 109 | 356 | 1,131 | 2,266 | 6,481 |
| F | I60-I69 |  |  | 2 | 2 | 20 | 85 | 257 | 944 | 4,088 |
| M | I60-I69 | 1 | 1 | 2 | 5 | 33 | 126 | 497 | 1,197 | 2,698 |
| F | I70 |  |  |  |  |  | 6 | 19 | 104 | 1,108 |
| M | I70 |  |  |  |  | 1 | 14 | 52 | 137 | 597 |
| F | I71 |  |  | 1 |  | 1 | 5 | 14 | 31 | 52 |
| M | I71 |  |  |  |  | 6 | 13 | 52 | 137 | 597 |
| F | I72-I78 |  |  |  |  |  |  | 4 | 22 | 109 |
| M | I72-I78 |  |  |  |  | 2 | 3 | 21 | 41 | 70 |
| F | J10-J11; J12-J18 | 797 | 3 | 3 | 2 | 9 | 21 | 51 | 133 | 563 |
| M | J10-J11; J12-J18 | 1,100 | 1 | 7 | 5 | 13 | 42 | 97 | 243 | 686 |
| F | J40-J42; J43 |  |  |  |  |  | 1 | 2 | 5 | 24 |
| M | J40-J42; J43 |  |  |  |  | 1 |  | 1 | 19 | 43 |
| F | J44 |  |  |  |  | 6 | 26 | 129 | 248 | 535 |
| M | J44 |  |  | 1 | 1 | 8 | 25 | 165 | 477 | 699 |

Source: Republic Fund for Health Insurance, authors’ calculations

**Table A6. Number of deaths caused by smoking and YWLL in Serbia in 2019 by age group**

|  | **35-39** | **40-44** | **45-49** | **50-54** | **55-59** | **60-64** | **35-65** | **65-75** | **75+** | **Total** |
| --- | --- | --- | --- | --- | --- | --- | --- | --- | --- | --- |
| Deaths | 57 | 162 | 292 | 622 | 1,316 | 2,301 | 4,751 | 5,017 | 6,553 |  |
| YWLL* | 1,604 | 3,733 | 5,258 | 8,086 | 10,531 | 6,904 | 36,117 |  |  |  |
| YRLL* |  |  |  |  |  |  | 47,510 | 21,224 | 7,089 | 75,823 |

Note: YWLL – Years of working life lost; YRLL – Years of retirement life lost

**Table A7.** **Share of illicit manufactured cigarettes (MC) and hand-rolled (HR) tobacco consumption in Serbia, 2019**

|  | Licit | | | Illicit | | |  |
| --- | --- | --- | --- | --- | --- | --- | --- |
|  | Average number of cigarettes smoked | Number of smokers | Total cigarette* consumption | Average number of cigarettes smoked | Number of smokers | Total cigarette consumption* | Illicit share** |
| MC | 17.3 | 2,224,728 | 38,452,392 | 15.9 | 59,387 | 943,244 | 2.4% |
| HR | 11.9 | 51,769 | 616,593 | 15.6 | 386,949 | 6,034,238 | 90.7% |
| Total |  |  | 39,068,986 |  | 446,335 | 6,977,482 | 15.2% |

Source: Đukić et al. (2021)

*Note: Total cigarette consumption is calculated by multiplying the average number of cigarettes smoked and the number of smokers. The average number of licit MC cigarettes smoked is 17.2840869 ̴ 17.3 The average number of licit HR cigarette smoked is 11.9104677 ̴ 11.9. The average number of illicit MC cigarette smoked is 15.8830047 ̴ 15.9 and the average number of illicit HR cigarette smoked is 15.5944013 ̴ 15.6

**The illicit share of 90.7% was derived from the study available at <https://tobaccotaxation.org/research.php?pID=254&lng=srb> and based on the survey STC-SEE 2019. Using the mentioned survey results, an HR illicit package is considered illicit source if at least one of the following applies: purchased from an illicit source; without the appropriate health warning; without the appropriate tax stamp.

**Table A8.** **Distribution of out-of-pocket medical expenses in Serbia by household income group, 2019**

| Income group | Select medical expenses* (RSD per HH monthly) | Select medical expenses as % of average medical expenses | Total income (RSD per HH monthly)** |
| --- | --- | --- | --- |
|  | (1) | (2) | (3) |
| Low | 1,980 | 83.4% | 55,905 |
| Middle | 2,303 | 97.1% | 65,749 |
| High | 2,837 | 119.5% | 79,688 |
| Average | 2,373 |  | 67,104 |

* Medicines, hospital services, outpatient care and inpatient care

** Total number of households is 2,466,316. Average household size is 2.68 people.

Source: HBS in Serbia, 2019

**Table A9.** **Distribution of out-of-pocket medical expenses in Serbia by household using RFSP data, 2019**

| Income group | Select medical expenses as % of average medical expenses | Select medical expenses* (RSD per HH monthly) |
| --- | --- | --- |
|  | (1) | (2) |
| Low | 83.4% | 5,775 |
| Middle | 97.1% | 6,725 |
| High | 119.5% | 8,275 |
| Average |  | 6,925 |

Source: The Republic Fund for Social Protection, Serbia

**Table A10. Tobacco expenditure, tobacco-related health costs, productivity and pension gains**

| **Income group** | **Part A**  **Tobacco expenditure**  **gains (%)** | **Part B Health costs**  **gains (%)** | **Part C Productivity  gains (%)** | **Part C+ Pension  gains (%)** |
| --- | --- | --- | --- | --- |
| Low income | 0.29 _[0.13, 0.44]_ | 0.82 _[0.71, 0.93]_ | 0.94 _[0.81, 1.06]_ | 0.83 _[0.72, 0.94]_ |
| Middle income | –0.19 _[–0.32, –0.05]_ | 0.48 _[0.37, 0.58]_ | 0.55 _[0.43, 0.67]_ | 0.49 _[0.38, 0.59]_ |
| High income | –0.52 _[–0.56, –0.49]_ | 0.17 _[0.14, 0.20]_ | 0.19 _[0.16, 0.23]_ | 0.17 _[0.14, 0.20]_ |

*Source*: Authors’ calculation. *Note*: Lower and upper bounds are displayed in superscript brackets

**Table A11.** **Change in disposable income by income group after tax increase, without pensions**

| **Income group** | **Lower bound elasticity** | **Middle bound elasticity** | **Upper bound elasticity** |
| --- | --- | --- | --- |
| Low income | 1.65% | 2.04% | 2.43% |
| Middle income | 0.48% | 0.84% | 1.19% |
| High income | -0.27% | -0.16% | -0.06% |

**Table A12.** **Change in disposable income by income group after tax increase, with pensions included**

| **Income group** | **Lower bound elasticity** | **Middle bound elasticity** | **Upper bound elasticity** |
| --- | --- | --- | --- |
| Low income | 2.38% | 2.87% | 3.37% |
| Middle income | 0.87% | 1.33% | 1.79% |
| High income | -0.13% | 0.01% | 0.14% |
